# Determination of Three Main Chlorogenic Acids in Water Extracts of Coffee Leaves by Liquid Chromatography Coupled to an Electrochemical Detector

**DOI:** 10.3390/antiox7100143

**Published:** 2018-10-15

**Authors:** Rocío Rodríguez-Gómez, Jérôme Vanheuverzwjin, Florence Souard, Cédric Delporte, Caroline Stevigny, Piet Stoffelen, Kris De Braekeleer, Jean-Michel Kauffmann

**Affiliations:** 1 Bioanalysis and Drug Discovery, RD3-Unit of Pharmacognosy, Faculty of Pharmacy, Université libre de Bruxelles, Campus Plaine CP 205/6, 1050 Brussels, Belgium; rrguezgom@ugr.es (R.R.-G.); Jerome.Vanheuverzwijn@ulb.ac.be (J.V.); florence.souard@univ-grenoble-alpes.fr (F.S.); cedric.delporte@ulb.ac.be (C.D.); Caroline.Stevigny@ulb.ac.be (C.S.); Kris.De.Braekeleer@ulb.ac.be (K.D.B.); 2 Department of Molecular Pharmacochemistry, Université de Grenoble Alpes, CNRS, DPM, 38000 Grenoble, France; 3 Analytical Platform, Faculty of Pharmacy, Université libre de Bruxelles, Campus Plaine, CP 205/05, 1050 Brussels, Belgium; 4 Botanic Garden Meise, Domein van Bouchout, Nieuwe laan 38, 1860 Meise, Belgium; piet.stoffelen@plantentuinmeise.be

**Keywords:** coffee, leaves, chlorogenic acids, chromatography, electrochemistry

## Abstract

Coffee is a beverage widely consumed in the world. The coffee species most commercialized worldwide are Arabica (*Coffea arabica)* and Robusta (*Coffea canephora)*. Roasted coffee beans are the most used, but coffee leaves are also consumed as infusion in several countries for traditional medicinal purposes. They contain several interesting phenolic antioxidant compounds mainly belonging to chlorogenic acids (CGAs). In the present work, a liquid chromatography-electrochemical detection (LC-EC) method was developed for the determination of three main chlorogenic acid isomers, namely 3-, 4-, and 5-caffeoylquinic acids (CQA), in coffee leaves aqueous extracts. Samples from eight coffee species, namely; *Coffea arabica*, *Coffea canephora*, *Coffea liberica*, *Coffea humilis*, *Coffea mannii*, *Coffea charrieriana*, *Coffea anthonyi*, and *Coffea liberica var. liberica*, were grown and collected in tropical greenhouses. Linearity of the calibration graphs was observed in the range from the limit of quantification to 1.0 × 10^−5^ M, with R^2^ equal to 99.9% in all cases. High sensitivity was achieved with a limit of detection of 1.0 × 10^−8^ M for 3-CQA and 5-CQA (i.e., 3.5 µg/L) and 2.0 × 10^−8^ M for 4-CQA (i.e., 7.1 µg/L). The chromatographic profile of the samples harvested for each *Coffea* species was studied comparatively. Obtained raw data were pretreated for baseline variations and shifts in retention times between the chromatographic profiles. Principal Component Analysis (PCA) was applied to the pretreated data. According to the results, three clusters of *Coffea* species were found. In the water sample extracts, 5-CQA appeared to be the major isomer, and some species contained a very low amount of CQAs. Fluctuations were observed depending on the *Coffea* species and harvesting period. Significant differences between January and July were noticed regarding CQAs content. The species with the best CQAs/caffeine ratio was identified. The LC-EC data were validated by liquid chromatography-high resolution mass spectrometry (LC-HRMS).

## 1. Introduction

Coffee trees belong to the genus *Coffea* in the Rubiaceae family. The genus *Coffea* L. comprises more than 100 species, of which only two species, that is, *Coffea arabica* (arabica coffee) and *Coffea canephora* (robusta coffee), are commercially cultivated on a large scale [1]. If the chemical composition of green coffee beans is well-known, less attention has been paid to the phytochemistry content of coffee leaves [2,3,4,5,6,7]. Interesting ethno-pharmacological uses and biological activities, which can partly be related to the presence of high amounts of chlorogenic acids (CGAs), have been described for coffee leaves [3,4,8,9]. Coffee leaves of *Coffea arabica* have been found to contain a total phenolic compound content at a concentration of 17.4 and 13.9% for young and mature leaves, respectively [10]. CGAs are present in coffee beans [4,11,12,13,14] and coffee silverskin [15]. Total chlorogenic acids content in coffee beans accounts for 5–10% of dry matter basis (dmb), which is a much larger amount than caffeine 1–2% dmb. The three principal (sub)groups of CGAs, i.e., caffeoylquinic acids (CQA), di-caffeoylquinic acids (diCQA), and feruloylquinic acids (FQA), represent approximately 67%, 20%, and 13% of total CGAs in Robusta, and 80%, 15%, and 5%, in Arabica leaves, respectively [12,16]. Chlorogenic acids in coffee beans are known to strongly influence the taste and the color of coffee extracts. Degradation of CGAs also contributes to the unpleasant complex taste found after prolonged extraction and beans roasting [11,17]. Antioxidant and antimicrobial properties of CGAs have been described and reviewed in the recent literature [13,18,19,20,21,22]. Several authors have reported that coffee polyphenols such as CGAs possess anticarcinogenic and antihypertensive properties, as well as hypoglycemic and hypolipidemic effects [4,22,23,24]. Coffee consumption has also been associated with high longevity and a low incidence of various degenerative and nondegenerative diseases in epidemiological studies [4]. Regarding the bioavailability of CGAs, approximately one third of the ingested amount via coffee consumption can be absorbed in the human gastrointestinal tract and metabolized [25,26]. As a food additive, chlorogenic acids exhibit antimicrobial activity and prevent the degradation of bioactive compounds. The double role of chlorogenic acids as nutraceutical and food additives makes such molecules excellent candidates for the formulation of dietary supplements and functional foods [23].

CGAs are phenolic compounds consisting of an ester formed from (-)-quinic acid and hydroxycinnamic acids, such as caffeic, ferulic, and p-coumaric acids. CGAs could be divided into several groups according to the type of ester substituent, namely, CQA = caffeoylquinic acids, FQAs = feruloylquinic acids, and p-CoQAs = p-coumaroylquinic acids. Three isomerizations of the quinic acid moiety are observed with ester formation at position 3, 4, or 5, such as 3-CQA, 4-CQA, and 5-CQA. The most abundant CGA in coffee plants and in some fruits, such as apples or blueberries, is 5-CQA (International Union of Pure and Applied Chemistry (IUPAC) numbering), also written 3-CQA in the pre-IUPAC numbering [27]. The major isomer in coffee brews was found to be the 5-CQA [28]. Biosynthesis of CGAs in the plant depends on several factors, such as changes in environmental conditions, plant stress, and pest infestation [29,30]. CGAs are secondary plant metabolites likely present in plants to protect against environmental stress. They appear to be associated with chloroplasts in very young coffee leaves and they accumulate in the leaf vascular system during leaf aging. Aged leaves have a CGA content ten times lower compared to young leaves [2,31].

Considering that drinking an infusion, prepared using coffee leaves, can be a source of CQAs, the present work aimed to develop a sensitive isocratic liquid chromatographic (LC) method for the quantification of three major CQA isomers in water extracts of *Coffea* leaves. It was also intended to provide a LC profile characteristic of the eight *Coffea* species investigated. The metabolic profile of these species in leaves was previously studied by liquid chromatography coupled to high-resolution mass spectrometry (LC-HRMS). It was noticed in the latter study that, except for *Coffea arabica*, no caffeine was detected (Limit of detection (LOD) below 0.01 µg/mL) in the water extract of mature leaves of the studied *Coffea* species [7]. This data was of special interest for the present study, since a high CGAs and low caffeine content in leaves or beans appear to be of importance when selecting a coffee extract for dietary beverage consumption [13].

Chlorogenic acids are readily oxidized in aqueous solution [32,33] and this property has been exploited for the assay of CGAs in coffees by differential pulse voltammetry [32]. It was shown that the electro-oxidation of the three CQAs and the three diCQAs occurred at approximately the same potential. Oxidation of the feruloylquinic acids (FQAs) occurred at more positive potentials. It was possible, by this method, to determine the total amount of CGAs in aqueous solution of different brands of coffees. The CQAs and diCQAs represented ca. 90–95% of all CGAs found and 5-CQA prevailed in all investigated coffee samples. The FQAs content was in the range 1–2% [32]. 

**Scheme 1:**
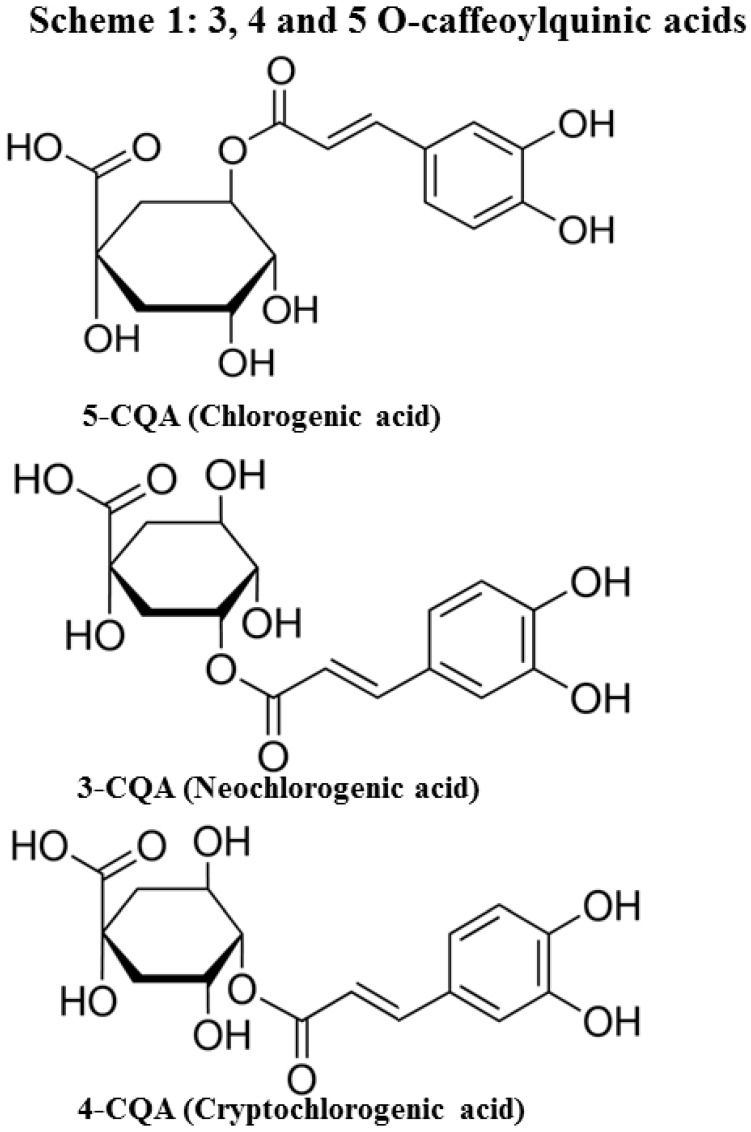
3, 4, and 5 O-caffeoylquinic acids.

In this work, the determination of three main CQAs in coffee leaves infusion was determined for the first time by liquid chromatography connected to an electrochemical detector (LC-EC) employing a boron-doped diamond working electrode (BDD). Compared to other detectors (UV-vis, mass spectrometry), an EC detector usually permits the achievement of a high sensitivity and recording of a chromatogram illustrative of readily oxidized CQAs, i.e., molecules with a high antioxidant activity [21]. The method was developed for the simultaneous determination of neo-chlorogenic acid (3-CQA), crypto-chlorogenic acid (4-CQA), and *n-*chlorogenic acid (5-CQA). The structure of the studied molecules is shown in Scheme 1 with CQAs IUPAC numbering. Eight coffee species were studied. They were grown in tropical greenhouses located in Brussels (Belgium) and the studied leaves were collected during two different periods: January and July 2016. The LC-EC data on CQAs content in the water extracts were confirmed by LC-HRMS.

## 2. Materials and Methods

### 2.1. Chemicals and Reagents

All reagents were of analytical grade, unless otherwise specified. Chlorogenic acid (3, 4, and 5-CQA) standards, mangiferin ((1S)-1,5-anhydro-1-(1,3,6,7-tetrahydroxy-9-oxo-9H-xanthen-2-yl)-d-glucitol), and sodium dihydrogen phosphate dihydrate were supplied by Sigma-Aldrich (Bornem, Belgium). Stock standard solutions of CQA (1 mg/mL) were prepared by exact weighing of approximately 10 mg of each compound into a 10 mL flask. Then, methanol (MeOH) up to the final volume was added. Working standard solutions for calibration and validation were prepared daily by appropriate dilution of stock standard solution with phosphate buffer prior to the experiments. MilliQ water was obtained from a Merck Millipore apparatus (Overijse, Belgium).

LC grade methanol, LC-Mass Spectrometry (LC-MS) grade acetonitrile, LC-MS grade formic acid (FA), and ortho-phosphoric acid 85% were obtained from VWR international Eurolab (Leuven, Belgium). Phosphate Buffer (PB) solution pH 3.5 consisted of a dihydrogen phosphate dihydrate solution 0.1M and the pH was adjusted with ortho-phosphoric acid 85%. MS quality trifluoroacetic acid (TFA) was purchased from Sigma-Aldrich (Steinheim, Germany).

### 2.2. Instrumentation and Software

The LC-EC set-up consisted of an LC pump 307 GILSON with a Rheodyne injection valve (20 μL injection loop) and an Atlantis^®^ C18 column (100 mm × 4.6 mm i.d., 3 μm particle size) from Waters (Elstree, UK). The amperometric detector was connected to a potentiostat Epsilon (BASi) (West Lafayette, IN, USA). The detector flow cell Flexcell^TM^ (Antec, Leyden, Netherlands) comprised a boron-doped diamond working electrode (Magic Diamond, Antec, Leyden, The Netherlands), a Pd/H_2_ (HyREF^TM^) reference electrode (REF) (Antec, Leyden, The Netherlands), and a carbon-loaded polytetrafluoroethylene (PTFE) auxiliary electrode (Antec, Leyden, The Netherlands). BASi® ChromGraph 2.51.001 control-USB 2.51.0225 software (BASi, West Lafayette, IN, USA) was used for instrument control, and ChromGraph^®^ 2.51.00.t REPORT 2.51.0425 software (BASi, West Lafayette, IN, USA) was used for peak detection and integration. All data analysis was performed by using Matlab version 2016b (The Mathworks, Natick, MA, USA). The algorithms for de-trending and Principal Component Analysis (PCA) were part of the ChemoAC toolbox (Freeware, ChemoAC Consortium, Brussels, Belgium, version 4.1) [34]. Peak alignment was realized using the algorithm for Correlation Optimized Warping (COW) [35,36,37,38]. Microsoft, Excell 2011 (Brussels, Belgium) was used for regression analyses. A pH-meter with a combined glass-Ag/AgCl (KCl 3 M) electrode from Metrohm (Antwerpen, Belgium) was used for pH measurements. Sample agitation was performed with a vortex-mixer (Vell, Brussels, Belgium). Dried leaves were ground into a fine powder using a mill. LC-HRMS analyses were performed using a 1200 series rapid resolution LC (RRLC) system coupled to a G6520A series electrospray ionization (ESI)-quadrupole time-of-flight (QTOF) high-resolution mass spectrometer from Agilent Technologies (Waldbronn, Germany). Data acquisition for LC-QTOF-MS analysis was carried out by MassHunter Acquisition^®^ software for QTOF (Version B.04 SP3), MassHunter Qualitative Analysis^®^ (Version B.06) software, and MassHunter Quantitative Analysis^®^ (Version B.04) software (Agilent Technologies, Waldbronn, Germany).

### 2.3. Sample Collection, Storage, and Extraction

A total of 24 leave samples from each of the seven different coffee species (*Coffea arabica*; *Coffea anthonyi*; *Coffea canephora*; *Coffea charrieriana*; *Coffea humilis*; *Coffea mannii*; *Coffea liberica*) and one subspecies (*Coffea liberica* var. *liberica*) were collected on 13 January and 28 July 2016, between 10:00 to 12:00 a.m.

All selected species, except *Coffea arabica*, are diploid and their natural habitat is the lowland rainforest of Central and West Africa. *Coffea arabica* is a tetraploid species from the highland rainforests from Southern Ethiopia. Six of the eight species selected are more or less closely related, including the two commercial species *Coffea arabica* and *Coffea canephora*, as illustrated in the most recent phylogeny of the genus [39]. Two species, namely *Coffea charrieriana* and *Coffea mannii*, have a more basal position in this phylogenetic tree and their seeds are reported to be caffeine free [39]. All sampled plants were more than 10 years old. To minimize environmental and climatic variations, all plants grew in the tropical greenhouses of the Botanic Garden Meise (Meise, Belgium) with identical environmental and edaphic conditions, such as natural daylight, substrate, watering regime, minimal temperature of 20 °C, and relative humidity of the air. The developmental stage of the leaves was categorized as mature leaves, defined by fully developed leaves and without any signs of aging or degradation (normally the second or third leave of a branch, counted from the top of the branch).

Leaves were manually collected and immediately put into hermetic bags containing silica gel, for water adsorption and preservation. The silica gel was replaced each day when necessary. Samples were dried and preserved for a minimum of seven days before the leaves were crushed using a grinder.

An aliquot of 15 mg of powdered leaves of a *Coffea* sample was suspended in 1.5 mL milli-Q water. The extraction procedure was carried out in water [7,29] and at room temperature. Heating was avoided in order to preserve the chemical structure of CQAs. This step was realized by sonication in an ultrasonic bath for 5 min at 55 kHz. Three replicates of each species of a coffee plant were prepared. The samples were filtered through a 0.2 μm cellulose acetate syringe filter and stored at −20 °C. Prior to analysis, 25 μL of each sample was collected and diluted to 1 mL in phosphate buffer pH 3.5 buffer.

### 2.4. CQA Quantification of Samples

Different concentrations of standard solutions (from 1.0 × 10^−8^ M to 1.0 × 10^−5^ M) were prepared and left to stand at 4 °C in the dark before analysis. The studied CQAs were quantified by both LC-EC and LC-QTOF-MS, by referring to a calibration curve prepared for each CQA. Peak identification and peak purity were performed by comparing the retention time with those of standards of the CQAs and by accurate molecular masses from LC-QTOF-MS analysis, respectively [7].

### 2.5. Liquid Chromatography-Electrochemical Detection (LC-EC)

The compounds were separated using an Atlantis^®^ C18 column in isocratic mode with a mobile phase consisting of 0.1 M phosphate buffer pH 3.5 with 15% of MeOH (*v*/*v*). Total run time was 25 min. The flow rate was 0.8 mL/min and the injection volume was fixed at 20 μL. The column temperature was maintained at room temperature. Electrochemical detection was performed by applying a potential of 1000 mV at the boron-doped diamond (BDD) working electrode. This detector was selected since besides innate properties of diamond, the BDD offers a wide electrochemical potential window in aqueous and non-aqueous media, low capacitive currents, a stable background, and weak adsorption of polar molecules in aqueous media [33]. Before use, the BDD was rinsed with methanol and gently dried with soft paper. The flow rate of the LC-EC set up was maintained overnight at 50 µL/min with the detector in the off position. Under such experimental conditions, the EC detector operated during six months of continuous use without sensitivity loss.

### 2.6. Liquid Chromatography Mass Spectrometric Detection (LC-QTOF-MS)

The chromatographic separation of targets analytes was performed using a Poroshell 120 EC-C18 column (100 mm × 2.1 mm i.d., 2.7 μm particle size). A gradient mobile phase was used with: 0.025% TFA + 0.075% FA (*v*/*v*) aqueous solution as solvent A and 0.025% TFA + 0.075% FA (*v*/*v*) in acetonitrile as solvent B. Gradient conditions were as follows: 0.0–8.0 min, 10% B; 8.0–9.0 min, 10–12.5% B; 9.0–11.0 min, 12.5–15% B; 11.0–17.0 min, 15–80% B; 17.0–18.0 min, 80–100% B; 18.0–19.0 min, 100% B and back to 0% B in 1.0 min and held for 8 min. Total run time was 28.0 min. Flow rate was 0.50 mL/min and the injection volume 10 μL. The column temperature was maintained at 55 °C.

Electrospray-ionization quadrupole time-of-flight mass spectrometry (ESI-QTOF) operated in positive mode with the following parameters: 2 GHz mode for resolution, mass range 100–1000 *m*/*z*, drying gas temperature 325 °C, flow 9 L/min, nebulizer pressure 55 psi, and capillary voltage −4000 V. Nitrogen served as the nebulizer and drying gas.

### 2.7. Chemometric Methods

In order to apply the chemometric data analysis tools, the chromatographic profiles were organized in a data matrix **X** (*n x p*), with *n* being the number of observations and *p* the variables (retention time t_R_).

### 2.8. Data Pretreatment

Baseline variations (parallel shifts and curvilinearity) and shifts in the retention time between the chromatographic profiles were noticed. Thus, a data matrix column (**X**) does not always contain equivalent information and this affected the outcome of the data analysis. Therefore, baseline correction and peak alignment or warping were applied. The baseline was corrected using the de-trending algorithm [34]. The baseline was modelled as a function of time with a second order polynomial and subtracted from the chromatographic profile. Correlation optimized warping (COW) was used for peak alignment [35,36,37]. The COW algorithm required the optimization of two input parameters by a trial and error approach [38].

## 3. Results and Discussion

### 3.1. Liquid Chromatography—Electrochemical Detection

A standard mixture of the three isomers CQAs at 3 mg/L (1.0 × 10^−5^ M) was investigated for LC-EC optimization. According to literature data, sodium dihydrogenophosphate-phosphoric acid buffer solution (0.1 M pH 3.5) was selected as the mobile phase [2]. Several buffers containing different amounts of MeOH (5, 10 and 15% *v*/*v*) were studied. With 5 and 10% MeOH, the retention time of CQAs was high (>50 min) and with MeOH at 15% *v*/*v*, the three isomers appeared at a retention time lower than 20 min. Other chromatographic parameters, such as flow rate and injection volume, were set at 0.8 mL/min and 20 µL, respectively.

**Figure 1:**
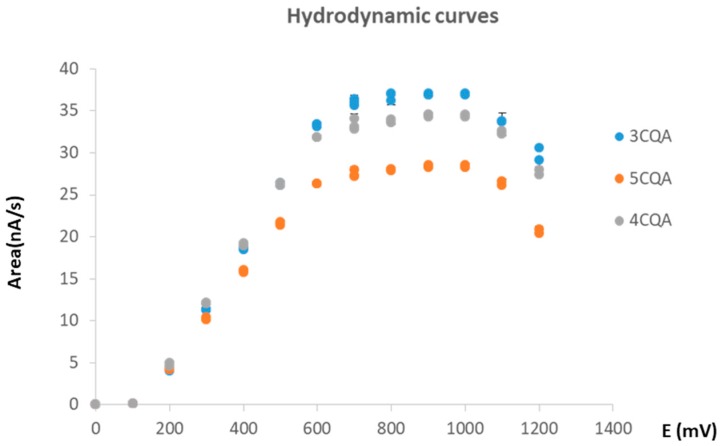
Hydrodynamic voltammograms. Studied potential from 0 to 1200 mV. Orange 5-*O*-caffeoylquinic acids (5-CQA), grey 4-CQA, and blue 3-CQA.

In order to optimize the applied potential, hydrodynamic curves for 3-, 4-, and 5-CQA were studied under the selected chromatographic conditions within the potential range of 0 to + 1200 mV. Figure 1 shows hydrodynamics curves for the three studied CQAs.

It appears that CQAs are readily oxidized compounds, with oxidation starting at approximately +100 mV, and the intensity of the signal increased until 900 mV, before stabilizing between 900 and 1000 mV. At 1100 mV, the signal started to decrease and the baseline was unstable. Due to these results, 1000 mV was selected as the optimum applied potential. It should be stated that at this potential, caffeine was not detected (not oxidized), and in the selected run time (25 min), the diCQAs and mangiferin, eventually present in the sample, were not yet eluted.

### 3.2. Analytical Performance

A calibration was realized for each CQA in LC-EC analysis. Seven concentration levels and three measurements at each concentration level were performed. The calibration graphs were constructed using peak area against concentration of the analyte. Table 1 shows the statistical and the analytical parameters obtained for each compound.

The LC-EC method was performed by considering linearity, sensitivity, precision, and selectivity criteria. A concentration range from the limit of quantification (LOQ) (see Table 1) to 1.0 × 10^−5^ M was selected. Linearity was expressed by the determination coefficient (% R^2^) and the *p*-value (% P_lof_) of the *lack-of-fit* test, for which values of 99.9 and higher than 5% were obtained, respectively (see Table 1). The minimum concentration of the analyte that the method can detect with a signal-to-noise ratio of 3 or 10 was used for LOD and LOQ determination, respectively. The values obtained for the LOQ ranged from 11.8 ng/mL to 23.7 ng/mL (Table 1). These LOQs compared favorably with the LOQ of 1.25 µg/mL and 2.2 µg/mL, as well as 0.06–0.4 µg/mL reported by LC-UV [12,14,28]. To evaluate the precision of the method, three replicates of each *Coffea* sample were injected in triplicate on the same day and on five different days. The precision was expressed as relative standard deviation (RSD). Intra-day repeatability was below 0.7% and inter-day repeatability was below 8% for the three studied CQAs. No interference from endogenous substances was found at the retention time of the studied CQAs in the majority of samples. This finding suggested that the electrochemical conditions ensure the selectivity of the method. However, in one of the studied samples, a strong interference was found in the LC-EC chromatogram. The interference was identified by LC-QTOF-MS (see below).

In order to confirm the results obtained by LC-EC, a calibration was realized for each CQA isomer by LC-QTOF-MS. Six concentration levels and three measurements at each concentration level were performed. The calibration plots were created by plotting peak area against concentration of the analyte. The LOQs obtained by LC-QTOF-MS were 1 µg/mL for 3- and 5-CQA, and 10 µg/mL for 4-CQA. 

**Figure 2:**
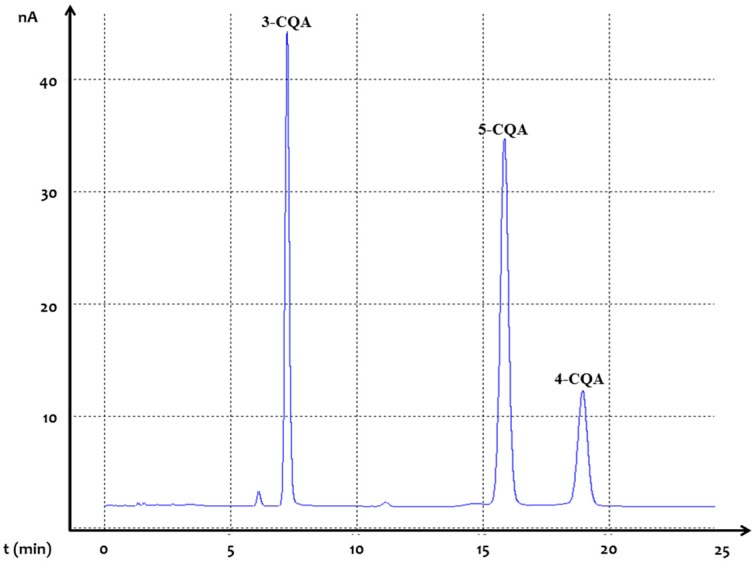
LC-EC chromatogram of a mixture of CQAs standard solution (3 mg/L). 3-CQA t_R_ = 7.5 min; 5-CQA t_R_ = 15.5 min; 4-CQA t_R_ = 19 min.

In the case of *Coffea liberica var. liberica*, a strong interference with the same retention time of 4-CQA was detected in LC-EC. This interference was confirmed by LC-QTOF-MS. By the latter method, a compound with 291.27 *m*/*z* [M + H]^+^ was found co-eluting with 4-CQA. According to this mass spectrum, epicatechin and catechin could be the most likely species. These molecules are often present in extracts from plants and typically in coffee leaves [40]. Under the present LC-EC experimental conditions, epicatechin eluted at a retention time longer than 20 min and it was present in most samples, but not in *Coffea liberica var. liberica*. Catechin, however, was found to be present in *Coffea liberica var. liberica* and it eluted at the same retention time as 4-CQA. Catechin is electroactive and this explained the big peak in *Coffea liberica var. liberica* at 19 min (4-CQA + catechin). Figure S1 shows the overlapped chromatograms (green line corresponds to catechin and blue line corresponds to *C. liberica var. liberica*). Figure 2 illustrates a typical chromatogram of standards with: 3-CQA t_R_ = 7.5 min (RSD = 0.2%), 5-CQA t_R_ = 15.5 min (RSD = 7.5%), and 4-CQA t_R_ = 19 min (RSD = 4.3%).

### 3.3. Chemometric Data Analysis

**Figure 3:**
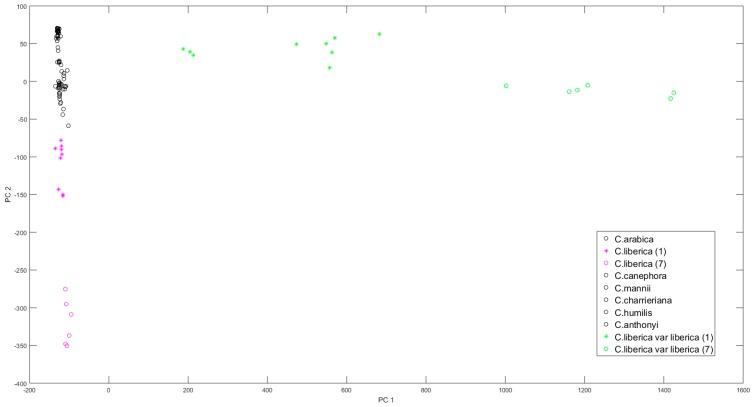
Score plot obtained after Principal Components Analysis (PCA) of the data matrix with all fingerprints due to species, and harvest period. PCA results after centering of dataset. Principal Component 1 (PC1) *versus* PC2; PC1 = 90.8% and PC2 = 6.8%. Label (1) indicates January and label (7) indicates July. Some species share the same symbol (*C. arabica*, *canephora*, *mannii*, *charrieriana*, *humilis*, *anthonyi*) and correspond to samples with a very low response and were thus not distinguished for better clarity of the figure.

Extracts of the coffee leaves, harvested in January and July, were analyzed by both LC-EC and LC-QTOF-MS for each of the eight coffee species studied. Interestingly, LC-EC only detected readily oxidized compounds and each chromatogram provided a unique “antioxidant profile”. PCA was applied to the data matrix with the baseline corrected and peak aligned fingerprints to test whether the chromatogram resulted in a clustering of the extracts. The score plots obtained after PCA are shown in Figure 3, as well as Figures S2 and S3.

In Figure 3, the PCA score plot exhibited two PCs, which explained more than 97% of the total variance (PC1 = 90.8% and PC2 = 6.8%). Three clearly separated clusters were obtained: one for *Coffea liberica* (pink area); one for *Coffea liberica var. liberica* (green area); and one for the other six species, i.e., low intensity profiles (black circles area)*.*

The loadings on PC1 showed the highest values at the retention time typical for 4-CQA. For *Coffea liberica var. liberica*, catechin was co-eluting with 4-CQA and dominated the fingerprint profile of this taxon. Catechin was not observed in the other fingerprints. PC1 was largely determined by this compound plus 4-CQA, since the time zone where these compounds eluted received the highest weight in determining the scores. 

The loadings on PC2 revealed the importance of the time zone where 5-CQA was eluting: PC2 was determined by 5-CQA. This explained why the fingerprint of *Coffea liberica* was clearly separated from the other taxa.

The score plot of PC3 vs PC1 was also represented, which explained more than 91% of the total variance (PC1 = 90.8% and PC3 = 0.7%), Figure S2 (see Supplementary data). The fingerprint for *Coffea humilis* was separated from the other species according to PC3. The presence of 3-CQA in the profile of *Coffea humilis* was attributed to the loadings on PC3 and therefore this antioxidant received the highest weight in the scores.

Figure S3 (see supplementary data) shows PC2 *vs* PC3, which represented more than 7% of the total variance (PC2 = 6.8% and PC3 = 0.7%). PC2 explained the variation in the “antioxidant profile” due to 5-CQA and PC3 due to 3-CQA.

Based on these data, *Coffea liberica* was separated from the other species according to PC2 because it contained the highest concentration of 5-CQA, and the “antioxidant profile” of *Coffea humilis* was determined by 3-CQA and was distinct from the other taxa according to PC3.

As mentioned above, the samples were collected in January and July. All plants grew in tropical greenhouses with the same environmental and edaphic conditions for the same month. Between months, however, exposure to sunlight, temperature, and relative humidity was different. In PCA plots, some differences between January and July could be found in the same species. The main differences between the harvest periods were observed for *Coffea liberica* and *Coffea liberica var. liberica*. Figure 3 shows two different clusters for *Coffea liberica*, one of them corresponding to January (labelled 1) and the other to July (labelled 7). This separation was due to the 5-CQA content. In January, the concentration was lower than in July (by approximatively 50%). In the case of *Coffea liberica var. liberica*, a similar situation occurred: two clearly separated clusters were found. The concentration from January to July was increased by approximatively 50% for 5-CQA and for the co-eluted peak (4-CQA and catechin). The other species gave peaks with a lower intensity, thus showing a less important “antioxidant profile”.

### 3.4. Application to Extracted Leaves

The LC-EC method was applied to determine the concentration of the three major chlorogenic acid isomers in water extracts. Eight coffee species were studied, namely: *Coffea arabica*, *Coffea canephora*, *Coffea liberica*, *Coffea humilis*, *Coffea mannii*, *Coffea charrieriana*, *Coffea anthonyi*, and *Coffea liberica var. liberica*. The LC-EC data were compared with respect to quantitative data obtained by LC-QTOF-MS (Table 2 and Table 3). The results obtained by both methods were comparable within the same order of magnitude. When CQAs are present, the 5-CQA appears to be the major isomer, in agreement with literature data for homemade brewed coffee samples [28], green coffee beans [14], and coffee leaves [31]. Its content was in the concentration range reported for coffee leaves [2,31] in which 5-CQA was found to represent about 90% of the total CGA amount [31]. There is a substantial difference in the studied species regarding the individual composition of CQA. Surprisingly, three species had a very low CQAs content, namely; *Coffea charrieriana*, *Coffea canephora*, and *Coffea mannii*. The variety *Coffea liberica* had the highest total content of CAQs in both January and July. Some seasonal variations were observed, with a general increase in total CQAs content in July. It must be emphasized that reported concentrations are equivalent to what is consumed as prepared from coffee leaves infusion prepared by local people. This data, however, cannot be reliably used to report absolute concentrations in samples unless the extraction efficiency is validated.

**Figure 4:**
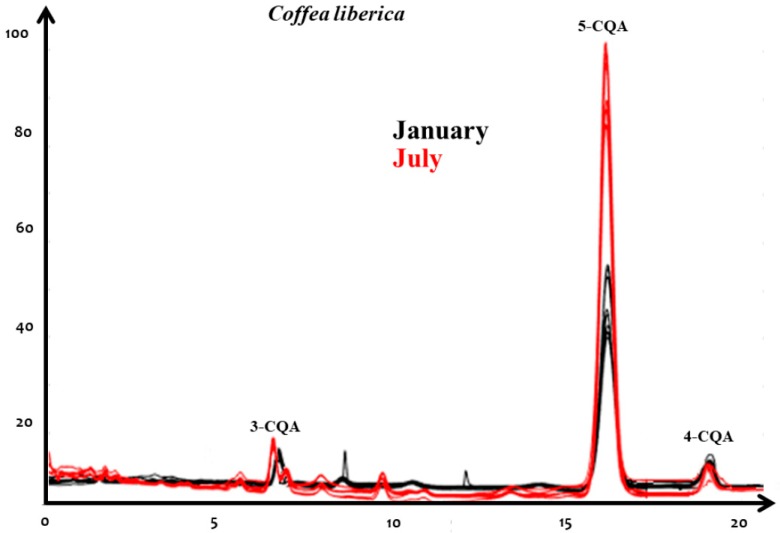
Typical LC-EC chromatograms of *Coffea liberica.* Identified peaks: 3-CQA, t_R_ = 7.5 min; 5-CQA, t_R_ = 15.5 min; and 4-CQA, t_R_ = 19 min. Red lines correspond to July and black lines correspond to January. Three extracts injected twice each.

Figure 4 shows typical LC-EC chromatograms obtained for the *Coffea liberica* species in January (black) and in July (red). The three main CQAs were present and an important increase in the content of 5-CQA can be observed in July compared to January.

In the case of *Coffea liberica var. liberica*, due to the presence of catechin at the retention time of 4-CQA, the latter could not be quantified by LC-EC. The presence of 4-CQA in *Coffea liberica var. liberica* was, however, confirmed by LC-QTOF-MS and the concentration was in the same range than the other species.

## 4. Conclusions

A new highly sensitive and readily implemented isocratic LC-EC method for the evaluation of the presence of three main chlorogenic acid isomers in a water extract of mature coffee leaves, prepared as a daily beverage drink, has been successfully developed. PCA on the baseline corrected and peak aligned chromatograms showed distinct clusters for each species. Notably, three CQA isomers appear to be key compounds for the discrimination of species. The different clusters were essentially governed by differences in each CQA content. In the same species, different clusters were obtained when January and July were represented together due to differences in the content of CQAs. With regard to quality criteria for beverage consumption, the present study showed a high content of CAQs in coffee leaves of some species. Data suggested the preferential selection of leaves from the *Coffea liberica* species collected in July due to the high total content in CQAs and very low (below LOD) caffeine content in the water extract. The present data have been obtained over a one-year period of study and further work over a longer period of time is needed in order to confirm the data. It should be pointed out that toxicological data are required before suggesting the consumption of coffee leaves infusions for medical purposes.

## Figures and Tables

**Table 1:** Analytical and statistical parameters.

Compound	(L∙A/s∙mol) ^b^	S_b_ (L∙A/s∙mol)	S_y/x_	% R^2^	LOD M	LOQ M	LDR M
5-CQA	1.27 × 10^10^	3.1 × 10^7^	2.88 × 10^2^	99.9	1.0 × 10^−8^ (3.5 ng mL^−1^)	3.3 × 10^−8^ (11.8 ng mL^−1^)	LOQ-1.0 × 10^−5^
4-CQA	7.54 × 10^9^	2.25 × 10^7^	2.15 × 10^2^	99.9	2.0 × 10^−8^ (7.1 ng mL^−1^)	6.6 × 10^−8^ (23.7 ng mL^−1^)	LOQ-1.0 × 10^−5^
3-CQA	3.87 × 10^10^	2.38 × 10^8^	2.21 × 10^3^	99.9	1.0 × 10^−8^ (3.5 ng mL^−1^)	3.3 × 10^−8^ (11.8 ng mL^−1^)	LOQ-1.0 × 10^−5^

^b^: slope; S_b:_ slope standard deviation; S_y/x_: regression standard deviation; R^2^: determination coefficient; LOD: limit of detection; LOQ: limit of quantification; LDR: linear dynamic range; CQA: caffeoylquinic acids.

**Table 2:** Content of the three main CQAs in coffee leaves extracts determined by Liquid Chromatography-Electrochemical Detection (LC-EC)

LC-EC								
JANUARY					JULY			
Found Amount (mg/L) ^a^								
Species	3-CQA	5-CQA	4-CQA	Total Amount	3-CQA	5-CQA	4-CQA	Total Amount
*Coffea arabica*	3.9 ± 0.05	41.0 ± 0.1	D	45.0	1.620 ± 0.005	37.0 ± 0.2	ND	39.0
*Coffea liberica*	3.90 ± 0.04	88.0 ± 0.5	4.20 ± 0.06	96.2	2.0 ± 0.2	190 ± 1	10.70 ± 0.06	203.0
*Coffea charrieriana*	ND	D	ND	-	2.170 ± 0.007	4.40 ± 0.02	D	6.6
*Coffea canephora*	2.10 ± 0.02	ND	ND	2.1	1.910 ± 0.004	ND	ND	1.9
*Coffea mannii*	1.00 ± 0.01	1.0 ± 0.2	ND	2.0	ND	2.40 ± 0.03	ND	2.4
*Coffea anthonyi*	3.50 ± 0.02	42.10 ± 0.04	ND	45.6	4.90 ± 0.04	43.0 ± 0.2	ND	48.0
*Coffea humilis*	12.0 ± 0.6	14.80 ± 0.06	ND	27.0	11.0 ± 0.2	29.0 ± 0.2	21.0 ± 0.2	31.0
*Coffea liberica var. liberica*	9.0 ± 0.1	49.30 ± 0.07	*	58.3	5.9 ± 0.04	118.0 ± 0.7	*	124.0

^a^ Mean of nine determinations; ND: not detected (<LOD); D: detected (>LOD and <LOQ). * No quantified due to presence of catechin in the sample.

**Table 3:** Content of the three main CQAs in coffee leaves extracts determined by Liquid Chromatography Mass Spectrometric Detection (LC-QTOF-MS).

LC-QTOF-MS								
JANUARY					JULY			
Found Amount (mg/L) ^b^								
Species	3-CQA	5-CQA	4-CQA	Total CQAs	3-CQA	5-CQA	4-CQA	Total CQAs
*Coffea arabica*	4.80 ± 0.07	36.0 ± 0.7	D	41.0	1.870 ± 0.003	26.60 ± 0.04	ND	28.5
*Coffea liberica*	9.40 ± 0.01	92.0 ± 0.7	D	101.4	8.70 ± 0.04	118.0 ± 0.1	ND	127.0
*Coffea charrieriana*	ND	ND	ND	-	3.10 ± 0.05	4.80 ± 0.02	ND	8.0
*Coffea canephora*	D	ND	ND	-	ND	ND	ND	-
*Coffea mannii*	1.10 ± 0.01	1.40 ± 0.02	ND	2.5	ND	1.80 ± 0.01	ND	1.8
*Coffea anthonyi*	4.20 ± 0.01	15.20 ± 0.07	ND	19.4	D	29.10 ± 0.04	ND	29.1
*Coffea humilis*	10.0 ± 0.4	13.50 ± 0.05	30.0 ± 0.2	53.5	18.0 ± 0.1	24.0 ± 0.2	17.0 ± 0.2	59.0
*Coffea liberica var. liberica*	1.040 ± 0.008	41.0 ± 0.3	D	42.0	10.0 ± 0.1	73.0 ± 0.4	14.30 ± 0.02	97.3

^b^ Mean of six determinations; ND: not detected (<LOD); D: detected (>LOD and <LOQ).

## Supplementary Materials

The following are available online at http://www.mdpi.com/2076-3921/7/10/143/s1, Figure S1: Typical LC-EC chromatogram of *Coffea liberica var. liberica* and LC-EC chromatogram of standard solution of catechin. Blue chromatogram corresponds to *Coffea liberica var*. *liberica* and green chromatogram corresponds to standard solution of catechin (1 × 10^−4^ M), Figure S2: Score plot obtained after PCA of the data matrix with all fingerprints due to species, and harvest period. PCA results after centering of dataset. PC1 versus PC3; PC1 = 90.3% and PC3 = 0.7%. Label (1) indicates January and label (7) indicates July, Figure S3: Score plot obtained after PCA of the data matrix with all fingerprints due to species, and harvest period. PCA results after centering of dataset. PC2 versus PC3. PC2 = 7.1% and PC3 = 0.7%. Label (1) indicates January and label (7) indicates July.
