# Supplementary material for: Determination of Three Main Chlorogenic Acids in Water Extracts of Coffee Leaves by Liquid Chromatography Coupled to an Electrochemical Detector
Source: Antioxidants (Basel). 2018 Oct 15;7(10):143. doi: 10.3390/antiox7100143 (PMC6209918; doi:10.3390/antiox7100143)
Supplement: Supplementary file 1 [file antioxidants-07-00143-s001.pdf]

Supplementary Material

Determination of three main chlorogenic acids in water extracts of coffee leaves by liquid chromatography coupled to an electrochemical detector

Rocío Rodríguez-Gómez[1], Jérôme Vanheuverzwjin[1], Florence Souard[1,2], Cédric Delporte[1,4], Caroline Stevigny[1], Piet Stoffelen[3], Kris De Braekeleer[1], Jean-Michel Kauffmann[1*]

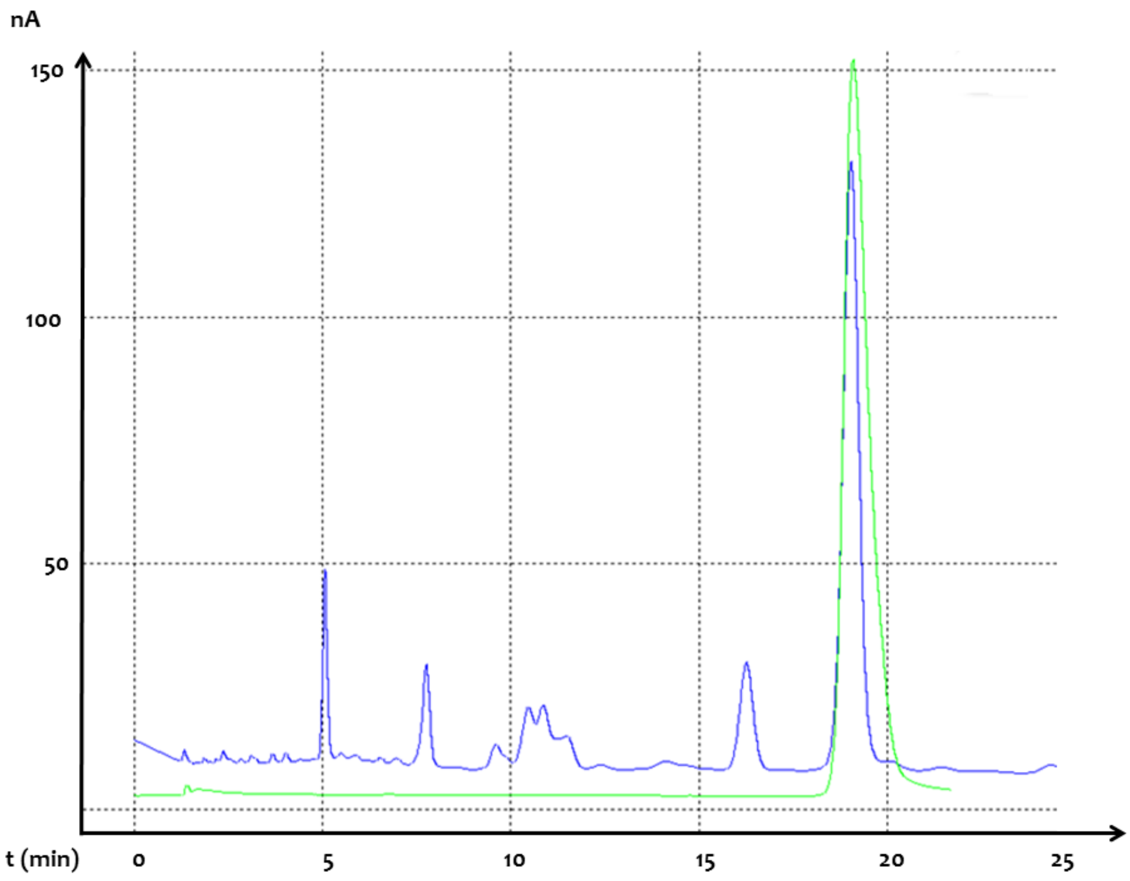

Fig. S1. Typical LC-EC chromatogram of *Coffea liberica var liberica* and LC-EC chromatogram of standard solution of catechin. Blue chromatogram corresponds to *C. liberica var. liberica* and green chromatogram corresponds to standard solution of catechin (1x10^{-4} M).

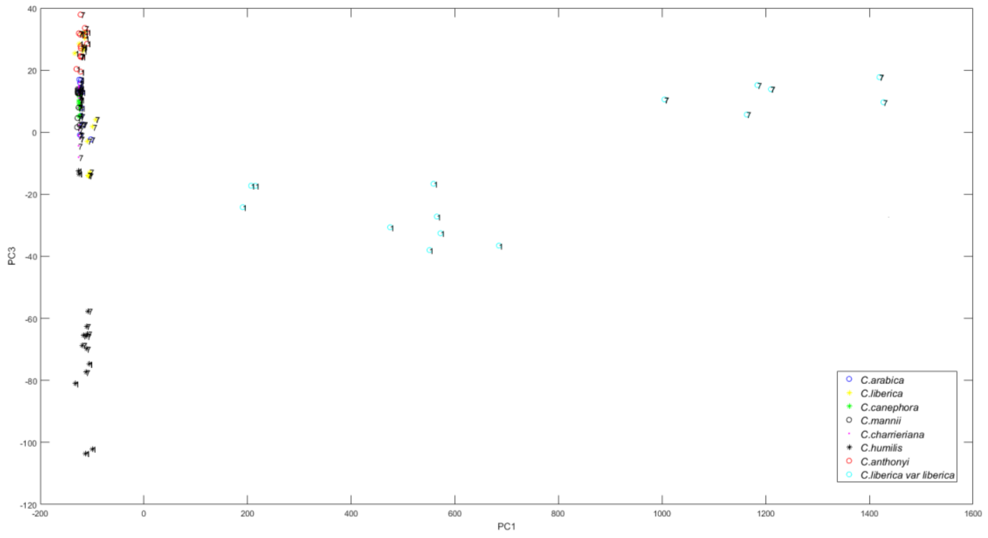

Fig. S2. Score plot obtained after PCA of the data matrix with all fingerprints due to species, and harvest period. PCA results after centering of dataset. PC1 *versus* PC3; PC1 = 90.3% and PC3 = 0.7%. Label (1) indicates January and label (7) indicates July.

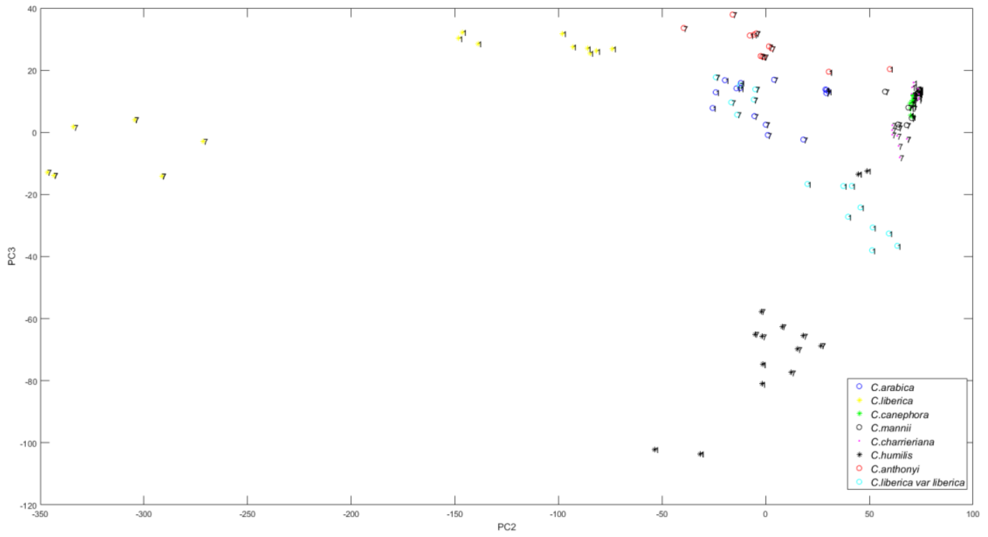

Fig. S3. Score plot obtained after PCA of the data matrix with all fingerprints due to species, and harvest period. PCA results after centering of dataset. PC2 *versus* PC3. PC2 = 7.1% and PC3 = 0.7%. Label (1) indicates January and label (7) indicates July.
